# Influence of gut and lung dysbiosis on lung cancer progression and their modulation as promising therapeutic targets: a comprehensive review

**DOI:** 10.1002/mco2.70018

**Published:** 2024-11-24

**Authors:** Rajan Thapa, Anjana Thapa Magar, Jesus Shrestha, Nisha Panth, Sobia Idrees, Tayyaba Sadaf, Saroj Bashyal, Bassma H. Elwakil, Vrashabh V. Sugandhi, Satish Rojekar, Ram Nikhate, Gaurav Gupta, Sachin Kumar Singh, Kamal Dua, Philip M Hansbro, Keshav Raj Paudel

**Affiliations:** ^1^ Department of Pharmacy, Universal college of medical sciences Tribhuvan University Bhairahawa Rupendehi Nepal; ^2^ Department of Medicine Kathmandu Medical College Teaching Hospital, Sinamangal Kathmandu Nepal; ^3^ School of Biomedical Engineering University of Technology Sydney Sydney New South Wales Australia; ^4^ Centre for Inflammation, Faculty of Science, School of Life Sciences Centenary Institute and University of Technology Sydney Sydney New South Wales Australia; ^5^ Department of Pharmacy, Manmohan Memorial Institute of Health Sciences Tribhuvan University, Soalteemode Kathmandu Nepal; ^6^ Department of Medical Laboratory Technology, Faculty of Applied Health Sciences Technology Pharos University in Alexandria Alexandria Egypt; ^7^ Department of pharmaceutical sciences, College of Pharmacy & Health Sciences St. John's University Queens New York USA; ^8^ Department of Pharmacological Sciences Icahn School of Medicine at Mount Sinai New York New York USA; ^9^ Department of Pharmaceutics Dattakala Shikshan Sanstha, Dattakala college of pharmacy (Affiliated to Savitribai Phule Pune university Pune Maharashtra India; ^10^ Centre for Global Health Research, Saveetha Medical College, Saveetha Institute of Medical and Technical Sciences Saveetha University Chennai India; ^11^ Centre of Medical and Bio‐allied Health Sciences Research Ajman University Ajman UAE; ^12^ School of Pharmaceutical Sciences Lovely Professional University Phagwara India; ^13^ Faculty of Health, Australian Research Centre in Complementary and Integrative Medicine University of Technology Sydney Ultimo New South Wales Australia; ^14^ Discipline of Pharmacy, Graduate School of Health University of Technology Sydney Ultimo New South Wales Australia

**Keywords:** dysbiosis, gut microbiome, lung cancer, lung microbiome, probiotics

## Abstract

Lung cancer (LC) continues to pose the highest mortality and exhibits a common prevalence among all types of cancer. The genetic interaction between human eukaryotes and microbial cells plays a vital role in orchestrating every physiological activity of the host. The dynamic crosstalk between gut and lung microbiomes and the gut–lung axis communication network has been widely accepted as promising factors influencing LC progression. The advent of the 16s rDNA sequencing technique has opened new horizons for elucidating the lung microbiome and its potential pathophysiological role in LC and other infectious lung diseases using a molecular approach. Numerous studies have reported the direct involvement of the host microbiome in lung tumorigenesis processes and their impact on current treatment strategies such as radiotherapy, chemotherapy, or immunotherapy. The genetic and metabolomic cross‐interaction, microbiome‐dependent host immune modulation, and the close association between microbiota composition and treatment outcomes strongly suggest that designing microbiome‐based treatment strategies and investigating new molecules targeting the common holobiome could offer potential alternatives to develop effective therapeutic principles for LC treatment. This review aims to highlight the interaction between the host and microbiome in LC progression and the possibility of manipulating altered microbiome ecology as therapeutic targets.

## Graphical abstract

Host–microbiome interaction in lung cancer. Both local (lung) and distal (gut) microbiota play critical roles in lung cancer

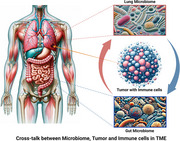

## 1 INTRODUCTION

Lung cancer (LC) has remained a global health challenge for a long time and is the most commonly occurring cancer type. World health organization (WHO) data showed that it had the highest prevalence rate among all cancer types, accounting for 12.4% of total newly diagnosed cases, and was the leading cause of death, consisting of 18.7% of all cancer deaths in the 2022 AD.^1^ LC has been reported to have diverse patterns of clinical manifestations, malignant features, and epigenetic alterations. Non‐small cell lung cancer (NSCLC) is the most predominant LC that constitutes 85% of total LC, and the remaining 15% of cases are categorized as small cell lung cancer (SCLC).^2^ Several therapeutic approaches, like radiotherapy, chemotherapy, surgery, targeted therapy, and immunotherapy, are currently being used for the treatments of LC, which are able to reduce its mortality rate.^3^, ^4^ Conventional strategies like surgical removal of tumor, radiation therapy (RT), and chemotherapy have improved the overall survival rate of treated patients.^5^ Continuous advancement in molecular biology and gene‐specific therapeutic innovation further make it possible to design personalized and disease‐specific treatment strategies like targeted therapy and immunotherapy that more precisely target tumor cells.^6^, ^7^, ^8^, ^9^ The research involving engineered exosomes,^10^ advance formulation/drug delivery using nanotechnology,^11^, ^12^, ^13^, ^14^, ^15^ decoy oligonucleotides,^16^, ^17^, ^18^, ^19^ microRNA inhibitors,^20^ and polyphenolic compounds^21^ are emerging as potential therapeutic targets for cancer, including LC. Pin‐point targeting of the key modulator in LC like epidermal growth factor receptor (EGFR), anaplastic lymphoma kinase, or ROS proto‐oncogene‐1 (ROS1) are promising features of some tumor‐specific therapeutic agents. Some of these novel agents work by modulating the immune response toward tumor cells and potentiating T cell's capability to attack cancer cells.^22^, ^23^, ^24^ Despite tremendous efforts and achievements in developing appropriate treatment of LC, it is still facing multiple obstacles and challenges because of therapeutic resistance develop by tumor cells.^25^ LC is highly vulnerable to epigenetic modulation and somatic mutation that induces resistance toward chemotherapeutic agents or immunotherapy, reducing their effectiveness and worsening disease prognosis.^26^, ^27^, ^28^, ^29^ WHO also takes this disease as a serious public health concern and has implemented dozens of policy initiatives with the hope of its early manifestations, reduce prevalence, improve treatment quality, and promote a healthy lifestyle. Strict administrative regulations related to tobacco trafficking, promotions and consumption, health awareness on tobacco‐based products, and advocating cancer‐free healthy society are some symbolic campaigns and strategic initiatives for mitigating LC prevalence.^30^, ^31^

The human microbiome forms a multifaceted interactive ecosystem among the external environment, microbiome, and host, especially with the host's immune system.^32^, ^33^ It collectively forms an integral host physiological system, and its transcription signal encodes proteins much more than the human genome itself.^34^, ^35^ Many human tissues and organs possess respective microbiomes and reveal specific features regarding population dynamics, species, and interspecies variability.^36^, ^37^ There are various structural and functional similarities and difference between gut and lung (Figure S1). Genomic interaction between host eukaryotic cells and microbiome prokaryotic cells revealed a dynamic and complex “holobiomic” philosophy, and this holobiont regulates all aspects of human physiology.^38^, ^39^ Microbiota has emerged as an essential component of the tumor microenvironment (TME) of most solid tumor.^40^ It is extensively reported as a dynamic player in carcinogenesis process, manipulating epigenetic sequence, DNA mutation, oncogenic pathway stimulation, and host immune activity modulation.^41^, ^42^, ^43^ The abundance of bacteria and composition of microbiome resides on specific tumor type have been found to be cancer specific. Further analysis revealed the close connotation between metabolic pathways of intratumoral bacteria and clinical feature.^44^ Many microbes produced various metabolic products that can cause DNA damage, alter cell cycle, and promotes the genomic instabilities leads to establish cancer cells more susceptible to mutagenicity.^45^, ^46^ Vast differences of bacterial taxa and microbiota composition between smoker and nonsmoker highlighted their crucial role in tumor modulation. Intracellular bacterial taxa from smoker showed the abundance of bacterium enriched with degradation pathway direct TME toward carcinogenic favor.^44^ Numerous studies have been published and reported that 1/5th of the total cancer cases globally are found to be closely associated with microbial infections like *Helicobacter pylori*, *Human papillsake omavirus, hepatitis‐B virus*, and *Epstein–Barr virus*.^47^ Several clinical cohorts demonstrated the meaningful correlation between compositional alteration of oral, lung, and gut microbiome and risk of LC prevalence.^48^, ^49^, ^50^ The active involvement of microbial secretions in inflammatory reactions and immune modulation of host immunity, along with their influence on enhancing the susceptibility of tumor oncogene toward mutation, provide new insights into microbiome engagement in carcinogenesis.^51^, ^52^, ^53^

Numerous cellular signaling pathways are actively involved in the modulation of LC progression and metastasis. RAS‐dependent mitogen‐activated protein kinase/extracellular signal‐regulated kinase (MAPK/ERK‐1/2) signaling, phosphatidylinositol 3‐kinase (PI3K)–protein kinase B (Akt)‐mediated cell proliferation and EGFR‐regulated ERK, and signal transducer and activator of transcription 3 (STAT3) pathways are the key regulating networks of NSCLC.^54^, ^55^, ^56^, ^57^ Multifactorial and heterogenic mutation of different genes that regulate and transcriptionally control cellular homeostasis, tweaking their cellular fate is the key factor of altered signaling events and initiation of cancer.^58^, ^59^ Inactivation of tumor suppressive function or adopting oncogenic function by p53 protein,^60^, ^61^ activation of Wnt 1 and Wnt 2 via Kirsten rat sarcoma viral oncogene homolog (KRAS) mutated transcriptional activity,^62^, ^63^, ^64^ triggering of STAT3 and ERK to stimulate their downstream signaling activation by mutated EGFR gene,^65^, ^66^, ^67^ upregulation of antiapoptotic Bcl‐2 and Bcl‐xL via constitutive overexpression of nuclear factor kappa B (NF‐κB) gene,^68^, ^69^ the oncogenic transformation of the normal cell by genetically altered Akt regulators,^70^ and inhibition of apoptosis by defunctionalization of Bad and MDM2 protein due to deregulated Akt/PKB transcriptional activity are the various transcriptional regulation abnormalities at the genetic level of LC biology.^71^, ^72^ Ras/ERK and PI3K/Akt/STAT3/NF‐κB are validated signaling cascades having promising therapeutic targets for LC management.^73^ Modulating tumorigenesis by specifically targeting single or multiple subsets of these signal cascade can help to improve the disease progression.^73^ Since phosphorylation and activation of various signal‐transducing intermediate substrates play crucial roles in cell proliferation and metastasis, kinase‐specific therapeutic agents are highly effective anticancer drugs.^73^, ^74^, ^75^ However, acquired resistance due to mutations at multiple targets of the EGFR sequence lowers their efficacy.^76^, ^77^ Considering all these obstacles, there is an urgent need for developing new therapeutic options that will have potency to correct the genetic manipulation in LC.^7^, ^78^

## 2 GUT MICROBIOME

**FIGURE 1:**
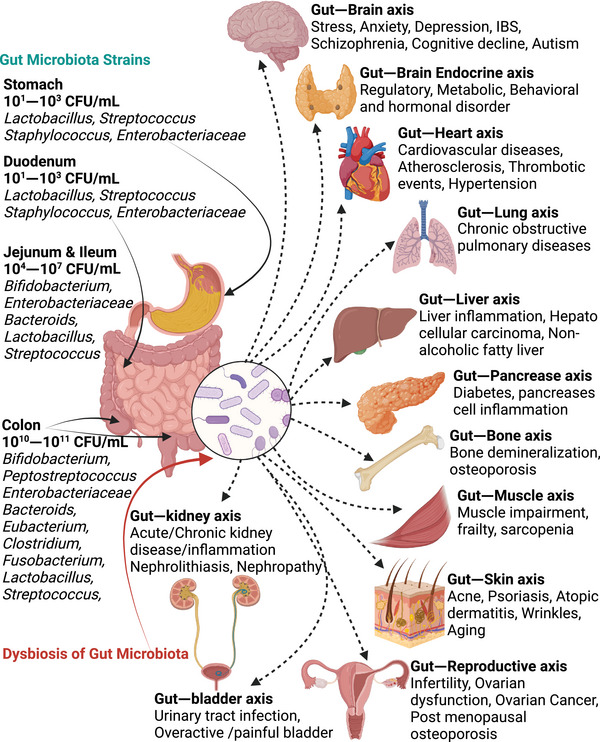
Gut microbiome species and impact of gut dysbiosis on human health. Gut constitutes well controlled and highly regulated microbial strain on healthy condition. Gut microbiota, either by their direct involvement or via metabolic products, dynamically regulate various organs of host. Dysbiosis of gut has been reported for their direct impact on health and functionality of most of the vital organs of human body. The bidirectional network that promises gut microbiome interaction to distant organs of body are gut–brain axis, gut–brain endocrine axis, gut–heart axis, gut–pancreas axis, gut–lung axis, gut–liver axis, gut–bone axis, gut–muscle axis, gut–skin axis, gut–reproductive axis, gut–kidney axis, and gut–bladder axis. The figure was reproduced with permission and slight modification (in BioRender) from Afzaal et al.^354^

It may be surprising that the cumulative population of microorganisms throughout our body system exceeds the total count of human cells. After the findings of the human microbiome project, the knowledge of normal flora has been extended dramatically and is advancing as an inevitable biological regulator of the normal human physiological system.^79^ The birth of any new human baby has been considered sterile in this world. But the presence of a hollow gastrointestinal tract (GIT) inside our body with two barrierless openings, that is, mouth and anus, into the external environment provide easy access for microorganisms from the microorganism‐dominated external world.^80^ Multiple organs and cellular sites of the human body are reported to have different types of microbiomes.^81^ Among all, the gut is the main reservoir of the human microbial ecosystem, providing the complete nutritional environment to survive and interspecies signaling among various kinds of microorganisms (Figure 1).^82^ The sterile gestational gut becomes colonized by diverse microorganisms and develops an individualized gut microbial composition that depends on the childbirth environment, feeding, and maternal genetic factors.^83^, ^84^ Human gut microbiota mainly comprises several bacterial phenotypes,^85^ predominantly *Firmicutes*, *Bacteroides*, *Proteobacteria*, and *Actinobacteria*.^86^ Additionally, several viral species, fungi, and archaea are also an essential components of gut microbiome,^87^, ^88^ and their phenotype is highly associated with an individual's diet pattern and gut bacteriophage composition.^89^, ^90^ Due to the anaerobic nature of the majority gut microorganisms, it is difficult to obtain them by in vitro culture technology. Only 1/3rd of the total discovered microorganisms was obtained in vitro using the different human serum‐based culture media. It needs to develop new microorganism‐specific culture conditioned technology to further explore the detailed molecular scope of gut microbiome.^91^

A strong anatomical, pathophysiological, and immunological cross‐talk exists between human host and gut microbiome.^92^ The gut microbiome, including all intestinal microorganisms, their metabolite, signaling molecules, cofactor, gene protein, and transcription factors plays a crucial role in the host's physiology.^93^ The gut microbiome can communicate with the host's body physiology mainly via the binding of pathogen‐associated molecular patterns with pattern recognition receptors primarily present in innate immune cell surfaces.^94^ The compactness of the outer layer of intestinal mucus largely depends on the type of microbiome present in the gut and the different proteases secreted by them. Commensal bacteria reside in the loose outer mucus layer and can easily utilize the glycan‐enriched mucin for their survival.^95^ Catalytic proteases produced by bacteria can metabolize the indigestible polysaccharides and glycan present in our intestinal mucin and generate energy. During this energy generation process, bacteria also produce different kinds of vitamins and short‐chain fatty acids (SCFAs) from carbon sources, which play a pivotal role in the maturation of the host immune system and intestinal epithelium.^96^ SCFA‐mediated activation of G‐protein coupled receptor (GPCR) present in gut epithelium surface and regulatory T‐cell (Treg) can induce the local and systemic effect of microbiota on the host.^97^, ^98^, ^99^ They can modulate the tolerogenic effect of dendritic cells and Treg, which ultimately inhibits the induction of allergic responses.^97^ A randomized controlled in vivo study highlighted the critical role of gut microbial homeostasis in immune cell regulation and chronic obstructive pulmonary disease (COPD) manifestations. Their investigation demonstrated that transferring fecal microbiota from healthy mice to a group of mice with cigarette smoke‐induced COPD rebuild the population of B cells, Ly6C monocytes, and CD8+ dendritic cells, resulting in improved disease prognosis.^100^ It is worth mentioning that absence of a gut microbiome leads to insufficient structural growth and immature gut lymphoid tissue, resulting in a weak gut immune system.^101^ Metabolic micromolecular byproducts and secondary metabolites of the gut microbiome have a positive affinity toward different epithelial cell surface receptors and exert their effect in the regulation of gut homeostasis and modulate different biological activities.^102^ Butyrate, the principal metabolic secretion of gut *Firmicutes*, is the major source of nutrition for continuously regerminating colon epithelium and enterocytes,^103^ while major metabolites of *Bacteroides*, i.e., propionate and acetate, are primarily utilized by hepatocyte and peripheral tissue, respectively.^104^ These SCFAs also exhibit immunomodulatory activity by generating different anti‐inflammatory signaling mechanisms like downregulation of NF‐κB pathway or promoting the chemotactic effect of neutrophils.^105^ SCFAs increase the expression of GPCR41 and GPCR43, which are associated with enhanced chemokines and cytokines release via MAPK signaling pathway inducing inflammatory and immune response in mice.^106^ Butyrate is well known for the downregulation of proinflammatory mediators IL‐12 and tumor necrosis factor (TNF)‐α. However, it upregulates the expression of various heat shock protein (HSP) like HSP 25 and HSP 72 in the intestinal epithelium.^107^, ^108^ This unique and complex gut ecosystem has a major impact on maintaining the host's immune homeostasis, and its functional role is not limited to only GIT. Rather, it acts as one of the important organ systems of the host.^109^, ^110^ Various microbiome population within gut and it influence on gut–other organ axis for disease progression is shown in Figure 1.

### 2.1 Gut dysbiosis and LC progression

**FIGURE 2:**
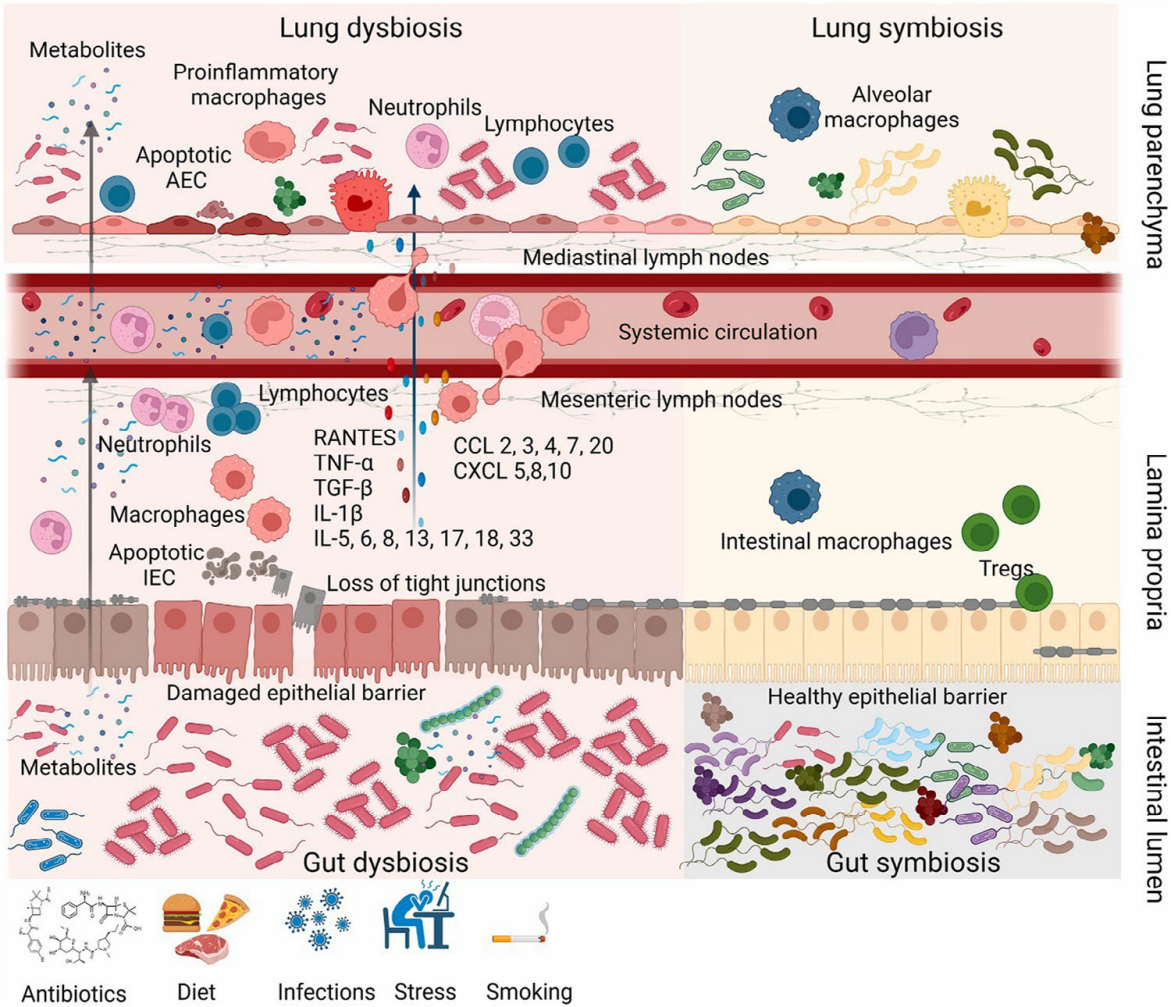
Outline of GLA and its material basis. Under symbiotic conditions, gut and lung's epithelial system comprises intact physiological barrier and maintain site‐specific microbial composition. Alveolar macrophages safeguard the lung tissue, while Treg and intestinal macrophages protect lumen lamina propia, together regulate the GLA homeostasis. Dieting habit, antimicrobial agents, various diseased conditions, lifestyle, and numerous environmental factors may lead to gut microbiota dysbiosis. Dysbiotic gut provokes the inflammation causing epithelial cell death, disrupts the compact epithelial barrier, and enhances intestinal permeability. Loose epithelial barrier provides easy access for normal flora, secondary metabolites, inflammatory cytokines such as TNF‐α, TGF‐β, IL‐1β, IL‐5, IL‐6, IL‐13, IL‐17, IL‐18, 1L‐10, and 1L‐30, other chemokines to systemic circulation. Also, various proinflammatory immune cells such as neutrophils and T‐cells can be recruited and induce lymphoid aggregation at gut mucosa, which subsequently get into systemic circulation and infiltered into distant organs including lung parenchyma. Additionally, mesenteric lymphatic system serves as significant way to translocate gut‐derived proinflammatory mediator to respiratory channel and stimulate alveolar macrophages and establish inflamed alveolar milieu. This imbalance causes alveolar epithelial cells apoptosis and alters alveolar barrier. Further, microbiome‐derived metabolites when reached the circulatory system, they can alter lung epithelial functions along with innate/adoptive immune response. This way inflammatory pathogenesis can mediate via GLA and alter lung physiology. Image was reproduced with permission (License no 5857010930891, dated August 27, 2024) from Eladham et al.^354^

Evidence of a strong correlation between gut microbiome composition and cancer progression has been well documented by many publications.^111^, ^112^ Circulation of soluble microbial content between gut and lung, increased plasma level of gut‐specific bacterial metabolites in pulmonary infection, significant alteration in gut microbiome composition in most of the pulmonary tuberculosis patients suggest that there should be an important physiological linkage between gut microbiome and respiratory pathophysiology.^113^, ^114^, ^115^, ^116^, ^117^, ^118^, ^119^ In fact, gut microbiota does not directly appear in the lungs. However, dynamic interaction between gut and lungs via a bidirectional GLA (gut–lung axis) allows the movement of different metabolites, hormones, endotoxins, and inflammatory mediators from gut microbiota. It is considered an important factor for different pathophysiological conditions of the lungs, including cancer.^120^ Evolutionarily, gut and lung share common embryonic origin and dynamic GLA pull these two distant organ systems into a closely linked and highly controlled microbial ecosystem to establish a common immunological cascade between them. Comparable mucosal coverage between gastric epithelium and alveolar tissue and other organ that constitutes the mucosal immune system foster the potential immune physiological dynamics between immune system and associated gut–lung microbiota via GLA.^121^ Existence of well‐controlled regulatory system between these organs through mesenteric lymphatic system and circulatory system attributes antigen‐specific systemic immunological responses; however extent of these responses primarily depends on site of antigen presentation.^122^ Exchange of nutrition and microbial metabolite's cross communication via GLA determine the overall status of microbiota, while high abundance of *Firmicutes* and *Bacteroides* attributes the major microbial population of healthy individuals. Though relationship between microbial population of lung and gut and their impact on different organ and health context is vague and needs to extensive investigation, it is obvious that antigen presenting dendritic cells, T cells, B cells, and intestinal epithelial cells are the commonly stimulated cells by GLA microbial communities.^85^, ^123^, ^124^ The dynamic physiology of GLA and its essence on human physiology can be further validated by understanding the impact of systemic sepsis on lung microbiome and gut microbiome. Acute lung injury (ALI) and acute respiratory distress syndrome (ARDS) are the common pulmonary complications associated with systemic sepsis.^125^ Sepsis mediated ALI and ARDS has been associated with disruption of alveolar bactericidal epithelial layer, exemplified the exudation of alveolar contents, induce oxygen gradients and upsurge the inflammatory cytokines that finally impaired the local immune responses. It can build a positive feedback loop of chronic inflammation and further advance dysbiosis.^126^ Similarly, it has been reported that sepsis can disbalance the homeostasis of claudin protein, a protein family responsible for intestinal mucosal integrity and maintenance of paracellular tight junction, led to hyperpermeability of intestinal barrier. This gut barrier interruptions make access of gut flora and their metabolite to systemic circulation and respiratory microvilli via blood and mesenteric lymph circulation.^127^, ^128^ The biomaterial basis of GLA now days advanced to secretion and regulation of 5‐hydroxytryptamine (5HT). Serotonin production by enterochromaffin cells of gut has been found to be controlled by transcriptional regulation of tryptamine hydroxylase 1, a major isoenzyme that limits the 5HT synthesis process from tryptamine. Secondary bile acid deoxycholate in response to spore‐forming microbiota and some Clostridium species shown the positive association with 5HT. Though exact mechanistic interplay between gut microbiome and 5HT is still yet to be elucidated, potential of microbial genome to synthesize 5HT has given a considerable insight to further explore the GLA scope on physiological basis.^129^ Emerging GLA concept and other microbiome research justify that gut–lung microbiome interaction conserves the major aspect of inflammatory reaction and mucosal immune response of the host's respiratory system.^130^ The essential material basis in the GLA is briefly summarized in Figure 2. This includes interaction of gut and lung microbiome with various cell in gut, lung, and immune cells in healthy state (lung and gut symbiosis) and various chemical (cytokines/chemokines) mediators released by these cells during damaged state (lung and gut dysbiosis).

Altered gut microbiome composition, commonly known as gut dysbiosis, is associated with dysregulated microbial metabolism and can alter the ideal biological composition of bacterial metabolites.^131^ Reduced abundance of beneficial microbiome and increased abundance of pathogenic microbiome has been commonly observed in various diseased condition. Dysbiosis has been identified with a different aspect of carcinogenesis, either in favoring tumor growth and diminishing anticancer treatment efficacy or assisting the antitumor response in some cases.^132^ LC patients without cachexia and other metabolic syndrome were enriched with commensal gut microbial species like *Eubacterium, Anaerostipes, Blautia*, and so on, as compared with patients with dysregulated metabolic syndrome.^131^ Skewed metabolic composition may progress with reduced production of butyrate and propionate that can be positively linked with DNA damage and cell cycle disruption, upregulate the carcinogens and potentially develop different types of genetically altered tumor cells.^133^, ^134^ Butyrate has been reported to induce apoptosis by inhibiting histone deacetylase and causes cell cycle arrest on G2/M phase, while propionate suppress the NSCLC aggressiveness by inducing chromatin remodulation via H3K27 acetylation and negative shift of epithelial to mesenchymal transitions.^135^, ^136^

Numerous gut microbiota and metabolites have been associated with systemic inflammatory cytokines recruitment and causes chronic pulmonary inflammation, a major cause of LC tumorigenesis.^137^, ^138^, ^139^ In particular, *Enterobacter* and *Escherichia shigella* have been found to be positively associated with systemic neutrophil‐to‐lymphocyte ratio, while *Dialister* demonstrated negative connection with neutrophile‐to‐lymphocyte or platelets‐to‐lymphocyte ratio, which are the potential predictive systemic inflammatory marker of LC.^140^ Similarly, *Enterobacteriaceae* cause the activation of intestinal Toll‐like receptor (TLR), upregulate IL‐1β expression in peripheral circulation, and encode inflammation to the lungs which ultimately stimulate NF‐κB activity and accelerate the pulmonary inflammations.^141^ Moreover, *Helicobacter* were strongly associated with IL‐6 production,^142^ while *Lachnospiraceae* and *Luminococcaceae* upregulated pulmonary TNF‐α and IL‐17.^143^ 12,13‐Dihydroxy‐9‐octadecenoic acid, metabolic byproduct of gut microbiota, has been indicated to stimulate lungs inflammations, repress the pulmonary Treg density, alter PARP‐γ‐regulated gene transcription in dendritic cells and inhibition of anti‐inflammatory cytokines secretions.^137^ Level of microbiota originated bile acid established the considerable association to numerous inflammatory markers like IL‐1β, IL‐6, and IL‐8.^144^ The progression rate of Lewis LC has been dramatically increased after antibiotic‐mediated induction of gut dysbiosis in mice model. Investigation at molecular level unveiled the reduced TNF‐α level on systemic circulation, suppression of intracellular adhesion molecule‐1, and diminish leukocyte movement toward tumor mass. The density of activated CD8^+^T cells was markedly reduced and Treg level was altered, which ultimately led to the altered immune environment on tumor region.^145^ Beside this, Dessein et al.^146^ clearly explained that gut dysbiosis extensively induces immunocompromised lungs and persistent suppression of cellular immunity in vivo. Authors demonstrated reduced hematopoietic cytokine Fms‐related tyrosine kinase 3 ligand, suppressed dendritic cell bone marrow progenitors, declined pulmonary macrophages, natural killer cells, neutrophils, and inflammatory monocytes.^146^ Clinical evaluation of enterotoxigenic *Bacteroides fragilis* and *Fusobacterium nucleatum* showed a noticeable increment in IL‐17, IL‐23, neutrophil levels, and potentially induced tumorigenic inflammatory tumor environment.^147^ Gut dysbiosis is associated with reduced responses toward several chemotherapy and immunotherapy‐based treatment regimens. Restoration of healthier microbiome composition after fecal transplantation from patients responding well to nonresponding patients became well responsive to anti‐PD1, CD8+ T cell activation.^148^ Abovementioned evidence strongly signifies the crucial association of gut dysbiosis in LC progression at host–microbiome cellular level.

## 3 LUNG MICROBIOME

**FIGURE 3:**
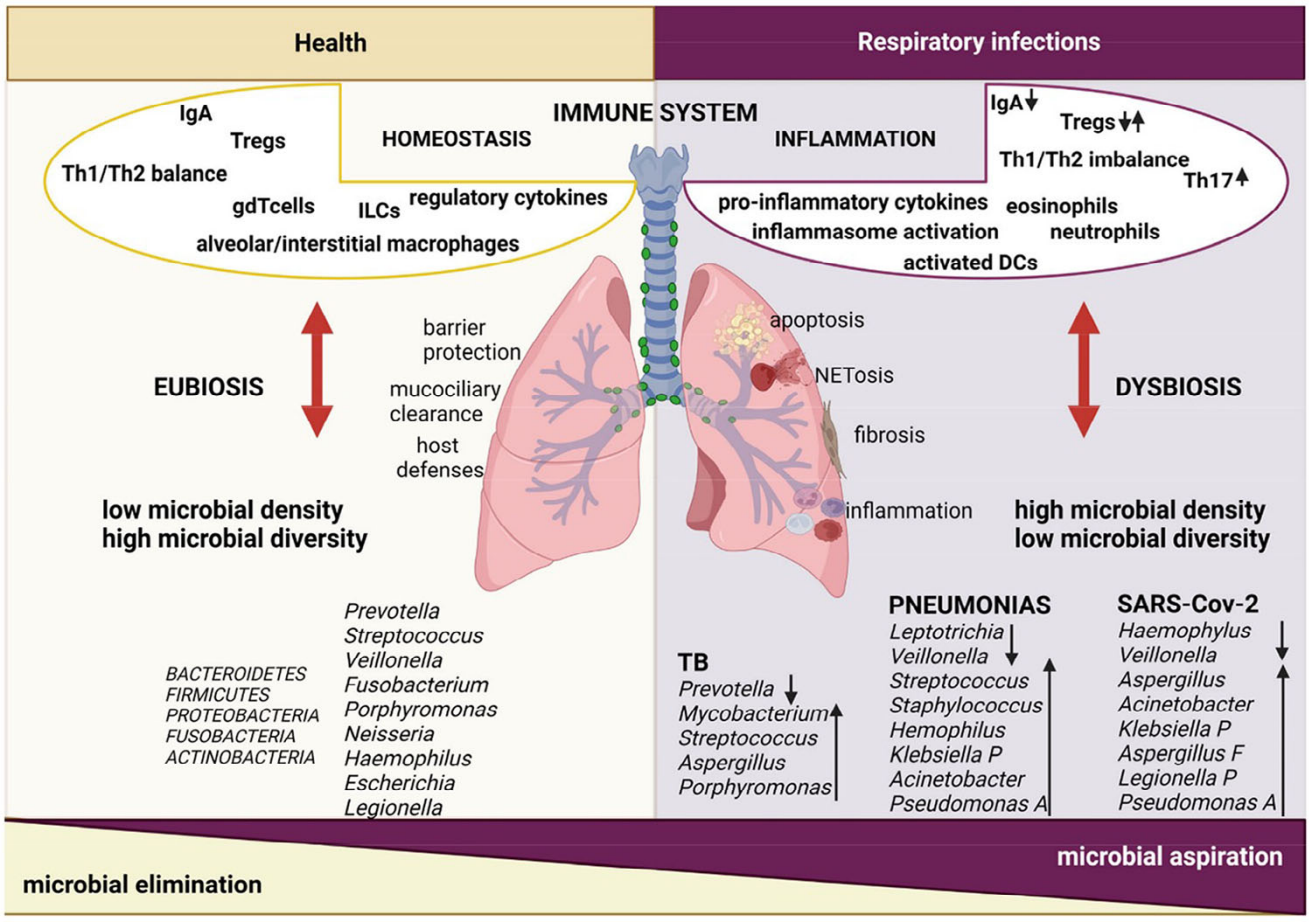
Composition of lung microbiome in health and diseased condition. Eubiotic lung comprises the higher abundance of *Proteobacteria, Firmicutes, Fusobacteria, Bacteroidetes*, and *Actinobacteria* family. They polarize naïve T cells, stimulate the maturation and differentiation of alveolar macrophages and Treg, sustain Th1/Th2 balance, and promote the local immune system homeostasis. However, when lung suffers with infectious disease, there is remarkable disruption on microbiome homeostasis; pathogenic and harmful microorganism oversite the lung tissue and cause lung dysbiosis. Then, translocation of immune cells to infected tissue promotes the secretion of pro‐inflammatory cytokines, activates inflammasome and DC, and induces inflammatory immune response. Altered cytokines mileu can promote lung tissue modeling and apoptosis.^356^

The contribution of the advanced research technique and emerging molecular approaches to understanding lung disease led modern health science to know its unsterile environment, which was considered sterile organs before.^149^ DNA‐based molecular sequence investigations played a vital role in exploring the compositions of existing microbial community inside the respiratory system. Instead of the conventional culture method that requires full nutritional media for microbial growth, this DNA‐based analysis utilizes the genetic quantification of 16s rDNA sequencing of bacterial DNA extract for extensive microbial elucidation.^150^ Various studies using this culture‐independent pyrosequencing technique proved the existence of a complex microbial ecosystem primarily dominated by *Prevotella, Veillonella*, and *Streptococcus* within the lower respiratory tract, including alveoli.^151^, ^152^, ^153^, ^154^ Though the primary source of microbiota for both the gut and lung microbiome is the oral microbiome, the micro‐anatomical characteristic of the respiratory system is quite different than GI system, irrespective of their same embryological origin.^80^, ^155^ Microbiome load in disease‐free lungs is 2–3 times lesser as compared with microbiome load of lower GI tract.^104^, ^154^ The food and microbial flow are unidirectional in normal gut physiology, while the movement of air, mucus, and microbes is bidirectional in the respiratory tract.^155^, ^156^ Also, the respiratory epithelium surface possesses a gradient environment from the ambient cool temperature at the air entrance zone to core body temperature at the alveolar level.^157^ Microbes entering the GIT from the oral cavity should be capable enough to cope up with both the acidic environment of the stomach and the basic environment of the small and large intestines.^80^, ^156^ The presence of abundant oxygen favors the more aerobic microorganism in the lungs. Fatty surface molecules that protect the alveoli have been reported to inhibit the growth and multiplication of some specific bacterial species like *E. coli, K. pneumoniae*, and *E. aerogenes* in the lungs.^158^ Moreover, the gut and lungs show different intraluminal and extraluminal macrophage responses toward inflammatory agents and thus have different host–microbe interactions.^159^ Regular air inhalation–exhalation phenomena allow several transient microorganisms in the respiratory tract, which also determine the composition of the lung microbiome.^160^ These factors makes respiration physiology much more complicated, and extensive dynamics resulting in complex lung microbiome composition. The composition of lung microbiome in both healthy and diseased condition is shown in Figure 3.

Microbial inhalation, excretion, and environmental growth conditions govern the microbial ecosystem and microbiome composition of the respiratory system. Significant alteration has been observed among these factors in most of the lung‐associated illness.^80^, ^161^ Respiratory environment with few microbial species is more prone to infections and other chronic disease as compared with diversified microbial species dominated environment.^162^, ^163^ Lung microbiota could behave as an oncogenic factor by promoting mucosal inflammation and immune imbalance.^164^

Overall, the healthy lung microbiome plays a crucial for regulating the lung environment and modulating immune responses to maintain homeostasis. It supports both innate and adaptive immunity by influencing the expression of immune‐related genes and promoting antimicrobial activities. Specifically, the lung microbiome supports both innate and adaptive immunity by upregulating PD‐1 expression while downregulating IL‐1α. Notably, it may enhance antimicrobial activity by activating macrophages through reactive oxygen species (ROS) production, inducing immune cells to produce cytokines like TNF‐α, IL‐6, IL‐10, and IL‐17, or by inhibiting TLR4 signaling. The adaptive immune response in the lungs plays a crucial role in disease progression, influencing the ecological balance of the microbiome.^165^ A number of studies have suggested that lung microbiome and lung dysbiosis can be a good target for focusing new research to develop better strategies to treat LC.

### 3.1 Lung dysbiosis and LC progression

Though only a few evidence have been known in earlier days, a recent study proved the existence of a strong association between the clinical pathophysiology of LC and lung microbiome (Figure S2).^166^, ^167^ Differences in microbiome composition between healthy and cancerous lung confirmed the role of lung dysbiosis in LC development. Existence of different microbial species in different LC conditions clarifies the role of specific microbiome in the progression of LC. Alpha diversity is significantly higher in nonmalignant lung tissue and lower in tumoral lung tissue, whereas beta diversity is almost similar in both tissues.^168^, ^169^ Critical risk factors for LC, such as continuous exposure to chemical carcinogens, cigarette smoke, environmental toxins, industrial/synthetic pollutants, toxic air particulates, chronic lung inflammatory diseases, lung fibrosis, and so on, can change lung microbiome composition, causing severe respiratory dysbiosis.^170^ Tobacco and cigarette smoking, the major causative factors of most LC,^171^ has been reported for its potential effect on the lung microbiome architecture, which then manifests to cause oral and respiratory dysbiosis.^152^, ^172^, ^173^ So that this mutated microbiome status is highly susceptible to further inflammatory process and take active participation in subsequent tumor progression to severe LC fatalism.^167^ This is also relevant to the type of coal used for cooking. Sputum from those who used smoky coal cooking and heating was present with lower alpha diversity.^174^ Huang et al.^175^ also established a valid correlation between lung microbiome composition with histopathology and the severity of tumorigenesis. They demonstrated that a much higher viable load of *Streptococcus* species was present in nonmetastatic lung adenocarcinoma (AC) compared with metastatic one.^175^ Similarly, *Veillonella* and *Rothia* were lower in nonmetastatic squamous cell carcinoma (SCC) as compared with metastatic SCC.^166^, ^175^, ^176^ Clinical investigation of samples from 216 LC patients demonstrated a significant increment of gram‐negative bacterial colon enriched with *Haemophilus influenza*, *Enterobacter*, and *Escherichia coli*.^177^ Moreover, remarkable changes in *Capnocytophaga, Selenomonas, Veillonella*, and *Neisseria* have been observed in salivary samples from AC and SCC patient compared with control patients.^178^ The proportion of *Firmicutes* to the *Bacteroides* is greater in smokers than in nonsmoker individuals, and the first group has a higher risk of LC occurrence.^50^

Comorbidity of the *Mycobacterium tuberculosis* (TB) infection and LC has been reported for a long time.^179^ Incidence of severe inflammation in most of the LC provide the new insight to investigate the presence of epidemiological linkage between these two deadly diseases.^180^ Chronic TB infection stimulates respiratory macrophages to produce TNF, which causes severe pulmonary inflammation and lung fibrosis. Extracellular matrix produced by fibrotic lungs play active role in initiation and progression of tumor. In another site, different tumor antigen, overexpressed oncoproteins, chemotherapy‐induced immunocompromised condition, and some radiotherapy cause the granulomas microenvironment deregulation, which favors the rapid multiplication of the *Mycobacterium tuberculosis* bacteria.^181^, ^182^ Two different meta‐analysis have suggested that LC incidence risk was increased in cases with previous chronic respiratory infection caused by bacteria, including tuberculosis, pneumonia, and *Chlamydia* pneumonia.^183^, ^184^ Prominent increment in *Saccharibacteria* (TM7) has been observed in COPD as well as cancer. These data might indicate that TM7 actively contributing to the development of cancer in most COPD patients.^50^ Yu et al.^168^ reported that only SCC was observed with more diverseness of bacterial phylogenesis along with elevated relative abundances of *Thermus* and decreased relative abundances of *Ralstonia* but AC did not. These data suggest that different microbiota might have different pathophysiological connections with proliferating cells in cancer histology. Additionally, high level of *Legionella* in metastatic cancer suggests that they could have an effective role in carcinogenesis process through different pathways.^168^ Observation of Mycoplasma, a key member of lung microbiota, in surgically removed LC tissue signify their active role in LC TME and indicates the strong association between mycoplasma infection and lung tumorigenesis.^185^ Similarly, *Streptococcus* are highly abundant in cancer cases, whereas *Staphylococcus* are highly abundant in normal cases,^186^ which is also supported by the study done by Lee et al.^50^ and Cameron et al.,^187^ indicating that changes in microbial composition of TME might be correlated with cancer development.

Any defects in symbiotic interaction between host immune sensing system and microbiome homeostasis generate a neural stress in their composition and initiate the translocation of various bacterial species, which ultimately leads host immunity to activate the antipathogenic response.^188^, ^189^ Persistent alteration in this phenomenon may exaggerate the microbiome dysbiosis and immune hyperreactivity. Other conditions such as inflammation at the respiratory site, pathogenic bacterial infection, individuals’ food and living attitude, disruption in circadian rhythm, and nutritional deficiency also may have a pivotal role in dysbiosis.^190^, ^191^, ^192^ Dysbiosis of microbiome reduces the commensal microbial load and shrinks their phylogenetic diversity but favors the accumulation of more pathogenic species inside host tissue.^193^ High incidence of pulmonary infection and postobstructive pneumonia and resultant poor LC prognosis indicates the existence of a potential link between microbiome and cancer progression.^194^, ^195^ Demonstration of tumor tissue with least abundance of *Staphylococcus* and *Dialister* compared with normal tissue and noncancerous tissue from LC patients suggests that lung dysbiosis is highly susceptible for progression of LC and its poor prognosis.^186^ A study done by Patnaik et al.^196^ reported that the reoccurrence of tumors after surgery can also be predicted based on the microbiota composition of the lower respiratory region. They reported the differential microbial richness at specific context of sample. Presurgical salivary sample of reoccurred group had double *Delftio* load and half *Bifidobacterium* density compared with the nonreoccurred group. Cancer biopsy showed significantly higher abundance of *Streptococcus* and reduced *Bacillus* or *Anerobacillus* level, while normal tissue were observed with consistent in both groups. Further, 16s RNA sequence analysis of BAL demonstrated increased density of *Sphingomonas*, *Psychromonas*, and *Serratia* and decreased *Calcibacterium*, *Geobacillus*, and *Brevibacterium* in tumor relapse group.^196^ Additionally, long‐term administration of antibiotics can also affect the normal microbiome composition and make patient more susceptible to cancer and another chronic disease. It was observed that the relative risk of LC was two to three folds more in subjects receiving more than 10 antibiotics as compared with the control population.^197^ A number of studies have been conducted to elucidate the exact mechanism of LC progression in lung dysbiosis. Bacterial metabolites or toxins mediated infection induced host inflammatory cellular signaling and host defense responses which may involve in tumorigenesis.^198^

As it has been evident that bacterial metabolite has direct impact on host cell metabolic processes and other signaling mechanisms,^199^ presence of metabolites from dysregulated microbiome in TME might affect cancer cell's metabolic pathway and other tumorigenic signaling.^200^ Suppression of KEGG module metabolism with elevated metabolism of amino acid, lipid, and xenobiotics has been dominated in lung microbiota of LC patients.^168^ This metabolic shift can further affect respiratory epithelium and alter their gene expression phenomena. Lung AC cell line treated with bacterial metabolite isolated from LC patient showed upregulated expression PI3K (an early event in cancer development) and ERK1/2 signal‐specific transcriptional gene, which follow the mutated transcriptional pattern similar to that observed in LC patient.^201^, ^202^ Additionally, manipulation of the alveolar airway by upper respiratory tract microbiota species like *Prevotella*, *Veillonella*, and *Streptococcus* resulted in the hypermetabolic activity of bacteria and upregulated the host mucosal immunity. Contrast to increased Th17/neutrophilic immune response, such hypermetabolic activity suppresses the innate immunity and potentially promotes the tumorigenesis via Th17/neutrophilic‐mediated immune modulatory mechanism.^203^, ^204^ In advanced knowledge, several evidence suggests that the local immune network of the respiratory axis is also influenced by microbiota residing in the lungs. Respiratory immune cells preserve the lung tissue homeostasis and induces the defense mechanism against pathogenic bacterial attack.^205^ Chronic inflammation is the key regulating mechanism of tumorigenesis and angiogenesis events of LC. Microbiome–host immune communication in dysbiosis causes infiltration of inflammatory cells that induces different proinflammatory factors such as cytokines, chemokines, and inflammatory prostaglandins, which provoke cell proliferation, angiogenesis, tissue remodeling, and metastasis.^206^ Dysregulated microbiota is associated with exaggeration of TLR‐dependent MyD88 signaling. TLR–myD88 coupling stimulates myeloid cells to release different kinds of interleukins like IL‐1β, IL‐17, IL‐22, and IL‐23 and promotes infiltration of neutrophils in TME. Phosphorylation of IL‐1 receptor‐associated kinases by MyD88 leads to upregulation of NF‐κB, MAPK, and activator protein‐1 signaling pathway that induces inflammation and promotes cell proliferation.^207^, ^208^ Enhanced Th17 lymphocyte level, inflammatory cytokines expression, and alveolar macrophages TLR4 responses during increased bacterial load of oral taxa in lower respiratory region indicate that respiratory microbiome composition play very crucial role in maintaining proper local immune homeostasis.^203^ Increased PDL‐1 expression in dendritic cells and T cell responses to adopt the TME when microbiome composition shifts from *Firmicutes* dominated to *Bacteroides* also supports the hypothesis of the essential role of lung microbiome in LC progression.^209^

Metabolites and toxins from pathogenic bacteria and their complementary cytokines released by immune cells can play an extensive role in LC progression by altering different cellular homeostatic mechanisms and another immune/inflammatory signaling pathway.^176^, ^210^, ^211^ Bacterial lipopolysaccharides (LPS) and lipoteichoic acid (LTA) can trigger host immune cells to produce proinflammatory mediators such as TNF‐α, IL‐1, and IL‐6 promoting chronic lung disease and induces LC.^212^, ^213^ LPS and LTA demonstrated integrin β3‐stimulated upregulation of PI3K–AKT–ERK1/2 pathway and promoted tumorigenesis progress in PC9 and H1299 LC cell line along with similar result in PC9 transfected nude tumoroid mice model.^214^ The effect of common microbiota in LC progression has been summarized in Table 1.

**TABLE 1:** Effect of common microbiota in lung cancer progression.

Microbiota types	Association with LC	Mechanism	References
*Streptococcus pneumoniae*	Positive	Stimulates cell proliferation by activating PI3K/AKT and NF‐κB signaling pathway via platelets activating factor receptor (PAFR)	^215^
*Veillonella parvula*	Positive	Reduce tumor‐associated T lymphocyte infiltration and activate Nod2/CCN4/NF‐κB signaling pathway	^216^
*Streptococcus* and *Veillonella*	Positive	Stimulate the activation of ERK and PI3K signaling pathway	^201^
*Cyanobacteria*	Positive	Microcystin from cyanobacterium reduced the CD36 and upregulates the PARP1 activity	^217^
*Acidovorex*	Positive	Promotes the transformed cell survival and helps in subsequent development of cancer; it causes TP53 mutation and DNA damage of pulmonary epithelia by its metabolic ROS/RNS.	^218^, ^219^
*Haemophilus*	Positive	Stimulate cell proliferation by upregulating IL‐17 and neutrophil infiltration; it can also increase the risk of metastasis in cigarette smokers.	^220^
*Lactobacillus rhamnosus GG*	Negative	Downregulates the expression of SNHG17 gene and control cell proliferations, differentiation, and tumor metastasis by inhibiting SNHG17/PTBP1/Nothch1 axis	^221^, ^222^
*Mycobacterium Tuberculosis*	Positive	Modulates host's immune responses as it strongly activates PD‐1 signaling pathway by stimulating expression of PD‐1, PD‐L1, and PD‐L2 on both CD4 and CD8 T‐cells	^223^
*Nocardiopsis exhalans*	Negative	It produces n‐(2‐hydroxyphenyl)‐2‐phenazonamine, which induces cytochrome *C* and Apaf‐1‐mediated caspase activation. Additionally, it also suppresses oncogene like IL‐8, TNFα, antiapoptotic protein Bcl2 and stimulates tumor suppressor gene P53 and P21.	^224^
*Stenotrophomonas maltophilia*	Positive	Enhanced A549 cells proliferation and migration by stimulating the histone deacetylase five gene expression	^225^

## 4 MODULATING MICROBIOME AS THERAPY FOR LC

The potentiality of microorganism‐based cancer treatment has a long history back, since the 19th century when a popular bone sarcoma surgeon, Dr William B. Coley, developed and applied different live and heat‐killed bacteria‐derived coley toxins and injected into patients with different types of cancer.^226^, ^227^ Despite the good efficacy of these coley toxins and prominent improvements in cancer treatment, his boss forced him to cancel all these projects by taking into consideration a few fatal cases that occurred at that time.^227^, ^228^, ^229^ But number of symbionts drew attention of researcher for their potential supportive role in cancer therapy especially in immunotherapy‐based treatment regimen. The biologically dynamic behavior of the host's microbiome with exogenous cytotoxic agents and immune modulators marked the human microbiome component as a core target of cancer therapy.^230^ For instance, reduced microbial diversity and enhanced Bacteroides due to broad‐spectrum antibiotics can lower the anticancer efficacy of oxaliplatin and cyclophosphamide by decreasing tumor infiltrating myeloid cell's reactivity with CpG‐oligonucleotides or by abolishing ROS generating ability of oxaliplatin.^231^, ^232^ Intriguingly, an interesting observation has been reported with irinotecan, a topoisomerase‐I inhibitor. Hepatic carboxylesterase‐mediated activation of irinotecan led to DNA breakage and cell cycle arrest, which ultimately directed cells toward apoptotic programming.^233^, ^234^ Hepatic uridine diphosphate–glucuronosyltransferase system catabolizes this active metabolite to produce inactive glucuronide form and secretes it on gut lumen for excretion.^235^ Unfortunately, bacterial β‐glucuronidase reactivate it again into active metabolic form thus exerting number of gut toxicities in later phase.^236^ Also, biological interaction established between the microbiome and host TME can directly inhibit tumor proliferation or produces immune‐mediated anticancer effects.^237^, ^238^ Polysaccharide‐rich ginseng has been found to increase antitumor response of αPD‐1 monoclonal antibody therapy by increasing abundance of *Parabacteroides distanosis* and *Bacteroides vulgatus* and then altering microbial metabolic processes. It increases valeric acid but decreases l‐kynurenine as well as kynurenine–tryptophan ratio which suppresses Treg and induces Teff cell after combined administration.^239^ Another study performed by Grenda et al.,^240^ found evidence that *Akkermansiaceae* bacteria, specifically *Akkermansia mucinphila*, were found to be highly supportive of improving the cancer therapy with immune checkpoint inhibitors (ICIs). Individuals with a higher abundance of *Akkermansiaceae* came out with better prognosis showing disease stabilization and partial immunotherapy response, but in contrast, a lower abundance of *Akkermansiaceae* in patients presented with continuous cancer progression.^240^ According to data from multicenter retrospective analysis, co‐administration of probiotics or postbiotics that helps to restore the gut microbiome homeostasis with different ICIs resulted in better clinical outcomes of NSCLC suggesting that probiotics can be a better choice for ICI treatment regimen.^241^ Routy et al.^242^ revealed that dysbiosis is one of the major hallmarks of the resistant of ICI to cancer cell. Their finding proved that fecal microbiota transplantation (FMT) from patients who are well responding toward PD‐1 blocking agent to germ‐free tumoroid mice model significantly ameliorated the antitumor effect of PD‐1 blockade whereas FMT from PD‐1 nonresponding patients failed to do so. Restoration of a balanced microbial ecosystem after the 30‐day administration of sodium butyrate that has been altered by chemotherapy done with paclitaxel extends the relationship between intestinal microbial ecology and cancer therapy regimen.^242^, ^243^

**FIGURE 4:**
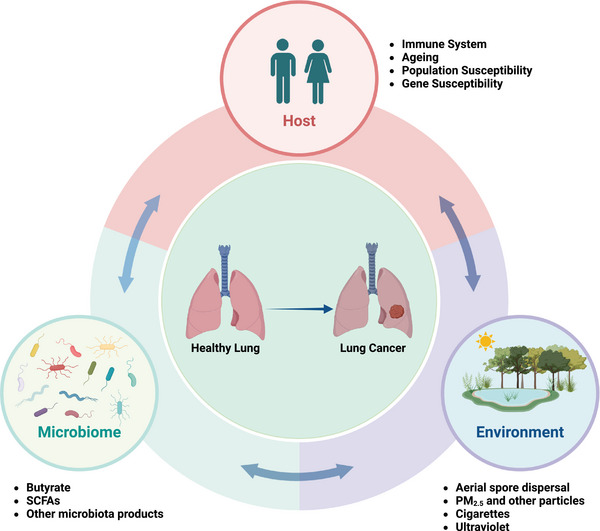
Ageing, population, or gene susceptibility are associated with tumorigenesis. As extrinsic factors, the microbiota produced the cytotoxicity‐related components, inducing the DNA damage of host cells. The microbiota and its metabolites (e.g., short‐chain fatty acids [SCFAs]) trigger downstream immune and metabolic signaling pathways, which further promote or suppress the malignant behaviors of host cells. Environmental factors (ultraviolet rays, cigarettes, and particles) can cause altered community of microbiota and gene mutations to promote the occurrence of lung cancer.

Catabolic metabolisms of major microbial metabolites are not only related to energy production but also regulate several signaling mechanism and immune responses that have a direct influence on the tumor cells. Xiao et al. showed that sodium butyrate significantly reduces A549 cell proliferation and arrests its metastasis by upregulating the TNF receptor‐associated factor‐6 (TRAF‐6)–thioredoxine interacting protein suggesting that sodium butyrate has potential antitumor effect in lung AC.^244^ A study independently done by Chen and Kim also proposed the potent role of butyrate and sodium propionate produced by microbiota in LC treatment in both in vitro and in vivo cancer models. These microbial metabolites were capable of modulating tumorigenesis by inhibiting cell proliferation and promoting apoptosis along with interruption of tumor cell metastasis. It decreases the cellular expression of antiapoptotic transcription factor KI67, CDK1, CDK2, survivin, and Bcl‐2 but upregulates apoptotic protein expression like Cyclin‐A, p21, Bax, and cleaved‐caspase3. Sodium propionate inhibited cell proliferation also by inducing cell cycle arrest specifically in G2/M phase of cell division.^245^, ^246^, ^247^ Similarly, a study conducted on clinical cohort of LC patients has found that patients having higher concentration of microbiota‐derived acetate on their body responded significantly better than patients having low acetate level.^248^ The host‐linked factors such as ageing, population, or gene susceptibility and environmental factors associated with tumorigenesis are shown in Figure 4.

**FIGURE 5:**
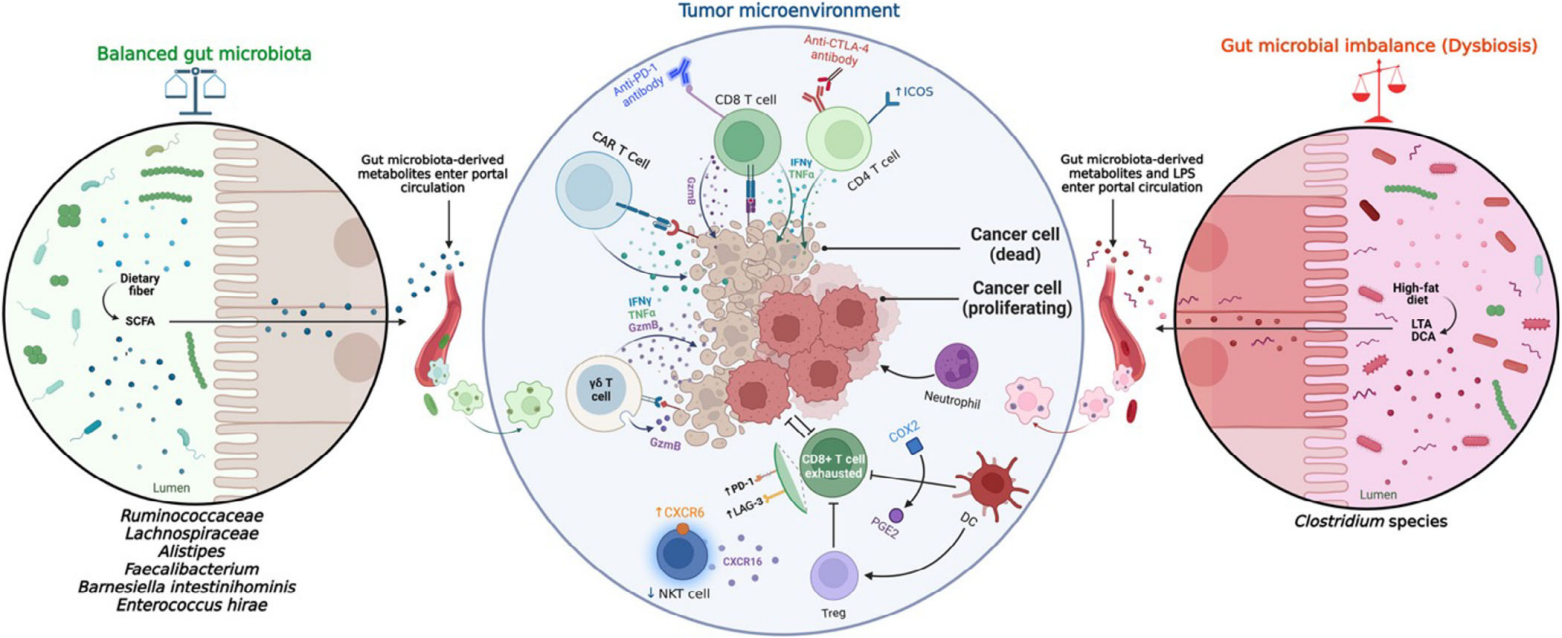
Impact of the gut microbiota on antitumor immunity. Crosstalk between host microbiome and immune cells may regulate cancer either in positive or negative way. Under properly maintained good gut microbiota and high fiber‐rich dietary condition, SCFAs obtained from beneficial flora translocate to systemic circulation and subsequently regulate T‐cell mediated response. SCFAs specifically facilitate the CD4+, ICOS+, CD8+ T cells accumulation, promote granzyme B, IFN‐γ, and TNF‐α expression, and strengthen the response toward immunotherapies like anti‐PD1/PD‐L1, anti‐CTLA4 antibodies, and CAR T cell therapy. SCFAs further stimulate IFN‐γ producing T‐cells on TME, collectively generate antitumor immunity. In contrast, gut dysbiosis enriched with harmful microbiome potentially upregulates BA production, which when enters to blood, triggers COX2 activation, enhances PGE2 synthesis, suppresses hepatic CXCL16, and diminishes NKT cells recruitments. These all contribute to tumor progression. Inhibition of CD103+ DC, accumulation of IFN‐γ, TNF‐α, and higher Treg presentation at TME help cancer cells tumor evasion. Overexpression of CD8+ T cells and severe chronic inflammation by dysbiotic flora cause T‐cells exhaustion and neutrophils recruitments that subsequently block antitumor immunity. This way, dysbiosis contributes to the cancer development. Image reproduced with permission from Mohseni et al.^357^

The lung microbiome presents a promising target for further investigation, offering potential in areas ranging from disease prevention and treatment to disease prediction, prognosis, and even LC therapy. The exact picture in the relationship between lung microbiota, immune system, and TME will be helping to develop the diagnostic LC biomarkers and new therapeutic strategies (Figure 5). The targeting specific bacterial species could enable the modulation of inflammatory responses, potentially creating a more antitumorigenic microenvironment. LC tumorigenesis is marked by an immune microenvironment that is rich in Th17 cell responses and characterized by the expression of IL‐17 and other cytokines. Additionally, lung commensal microbiota dominated by upper respiratory tract microbes contributes to a Th17/neutrophilic phenotype within the lung microenvironment. Consequently, it can be inferred that lung microbiota enriched with URT microbes promotes a protumorigenic microenvironment by inducing Th17 responses. BPs could be engineered to target specific lung microbiota, potentially altering the tumor and immune microenvironment in LC patients to promote a more antitumorigenic environment. Modulating the lung microbiota in LC patients could significantly impact tumor growth and progression by reshaping the immune microenvironment. The clinical development of various targeted drugs can impact the local TME. For instance, these drugs can directly target the microbes, their metabolites, phages, or induced cytokines (e.g., IL‐17) to reshape the microbiome, thereby reducing tumorigenesis or slowing tumor progression.^165^, ^249^

### 4.1 Natural compound exhibit microbiome mediated anticancer activity

Many biologically active natural compounds like alkaloids, flavonoids, saponins, polysaccharides, and other herbal molecule have emerged nowadays as the best targets for anticancer therapy development due to their better safety profile and potential cytotoxic effect.^250^ So many traditional medicinal compounds isolated from natural sources exert cancer protective and healing properties by regulation of gut microbiome and thus regulate the host immune system, inflammatory responses, angiogenesis, and tumorigenesis.^251^, ^252^, ^253^, ^254^, ^255^ Several studies have been conducted to revealed the effect of *Polygonatum sibiricum* polysaccharides (PSP), water‐insoluble polysaccharides (WIPs), Astragalus polysaccharides, Astragalus mongholicus polysaccharides, cordyceps sinensis polysaccharides, and turmeric polysaccharides in reconstruction of gut microbiota composition and to discover cellular signaling mechanism to exert physiological function in host physiological system.^256^, ^257^, ^258^, ^259^, ^260^, ^261^ Luo et al.^256^ demonstrated that PSP reduced Helicobacter abundance and enhanced intestinal abundance of *Akkermansia* and proposed that PSP can improve the inflammatory environment by reducing amyloid‐β accumulation. WIPs obtained from *Poria cocos* mushroom markedly stimulates the colonization of butyrate producing *Lachnospiraceae* and *Clostridium* bacteria and upregulates PPARγ signaling pathway to regulate cellular proliferation and differentiation.^257^ Gong et al.^252^ introduced evidence that saponins from *Astragalus* have antitumor, antihypertensive, antidiabetic, and lipid‐lowering properties and human‐immunity improving effects. When injected via the IV route, it reconstructs gut flora and regulates AMPK/SIRT1 or PI3K/AKT pathway, thus can be a potential target of antitumor therapy. A triterpenoid saponin glycoside from the root of liquorice, namely, glycyrrhizic acid (GA), has been known for its magical antitumor and antimetastatic properties. Glycyrrhizin disrupts the lung tumor invasion and migration by downregulating high mobility group box‐1 transcription factor. GA regulates gut microbiome and modulate its effect on host cell immune responses by suppressing M1‐like colonic macrophages, and it inhibits the formation of tumor and premetastatic niches via downregulation of LPS/HMGB1/NF‐κB signaling. The gut microbiome regulation has been easily determined as marked reduction in *Clostridiales* order and *Desulfovibrio* genus that ultimately reflected as reduced *Firmicutes* to *Bacteroidetes* ratio.^262^, ^263^ Similarly, Lu et al.^256^ demonstrated that a polysaccharide derived from Spirulina significantly reduces lung tumor volume and arrest cancer progression in tumoroid mice model by regulating arachidonic acid metabolism. After gut microbiome sequence analysis, it has been revealed that it restored gut flora homeostasis by increasing *Lactobacillus, Alloprevotella, Allobaculum*, and *Olsenella* abundance with decreased Bacteroides and Actinobacteria.^256^ All these evidences reveal the direct impact of the herbal‐based compounds and other natural formulations on a different points of LC pathogenesis via microbiome‐modulated cell death signaling and immunomodulatory ability, attributes a new horizon for anticancer research and finding of better integrative treatment strategies for LC.

### 4.2 RT and microbiome

RT emerged as a highly effective therapeutic strategy for most of the LC types, and it has been observed that every patient should go for RT at least once during the total course of disease.^264^, ^265^, ^266^ High prevalence of respiratory pneumonia and pulmonary fibrosis remains the most common and challenging RT‐induced complications in patients suffering from chest cancer, especially LC and breast cancer.^267^ In the beginning, RT utilized the precise and specifically optimized ionizing radiation (IR) from different sources that have been directly applied to cancer cells.^268^ To minimize IR‐associated adverse event, rigorous research and technological approaches translated the conventional radiation protocol and advanced to development of internal delivery of IR like brachytherapy, local implantation of specific radioactive materials to tumor site, systemic delivery of tumor‐specific radiation, and receptor‐specific radioactive pharmaceuticals.^269^, ^270^ Prevalence and severity of RT adverse event mainly depends on the dose of IR and targeted cell/organ's sensitivity toward RT.^271^ The human gut is reported to be comparatively more susceptible to IR than others.^272^, ^273^ Interestingly, gut microbiome has been found to be highly radiosensitive and their composition determined the radiosensitivity of host's cell also.^274^, ^275^ The low survival rate of normal mice when exposed to the same dose of total body irradiation applied to GF mice further validates this hypothesis.^276^ Gut microbiota possesses different extents of sensitivity toward IR, and it is inevitable to protect them from IR exposure during RT treatment. Depends on dose and exposure time, IR directly alters the qualitative and quantitative characteristics of gut microbiome ecosystem, which are emerging as important novel biomarker of radiation exposure and IR dose adjustment. This dynamic interaction is always functions in bidirectional way as RT disrupts the microbiome population and disrupted status of microbiome greatly influence the effectiveness of RT treatments. Gut dysbiosis and microbiota translocation are the two major mechanistic interplay to measure the RT effectiveness and potential side effect.^275^, ^277^, ^278^ Relative reduction of the commensal microbiome and increased harmful species in a correspondent way establishes transitional gut dysbiosis and ultimately reduces commensal flora synthesized SCFAs in host biology.^278^ Decreased *Firmicutes* microbial load, increased *Bacteroidetes* counts, decrease in alpha diversity, and increase in beta diversity are common findings associated with IR therapy.^279^, ^280^ 16S rRNA sequence analysis of sample from irradiated large intestine has been observed with increase in *Verrucomicrobia* phyla in contrast to decreased level of *Prevotella* and *Mucispirillum*.^281^ Consequences of altered *Firmicutes* to *Bacteroidetes* ratio after RT not only confined to undesirable outcomes of irradiation therapy. Rather, it can also weaken the intestinal epithelial barrier, allow microbiota to be translocated into deeper tissue lesions, and assist microorganism and their metabolites to reach the systemic circulation.^271^, ^282^ Observation of different systemic inflammatory markers such as interleukin‐1β, IL‐6, and TNFα in both clinical samples and animal models after irradiation therapy further proved this systemic effect of RT.^283^, ^284^

**FIGURE 6:**
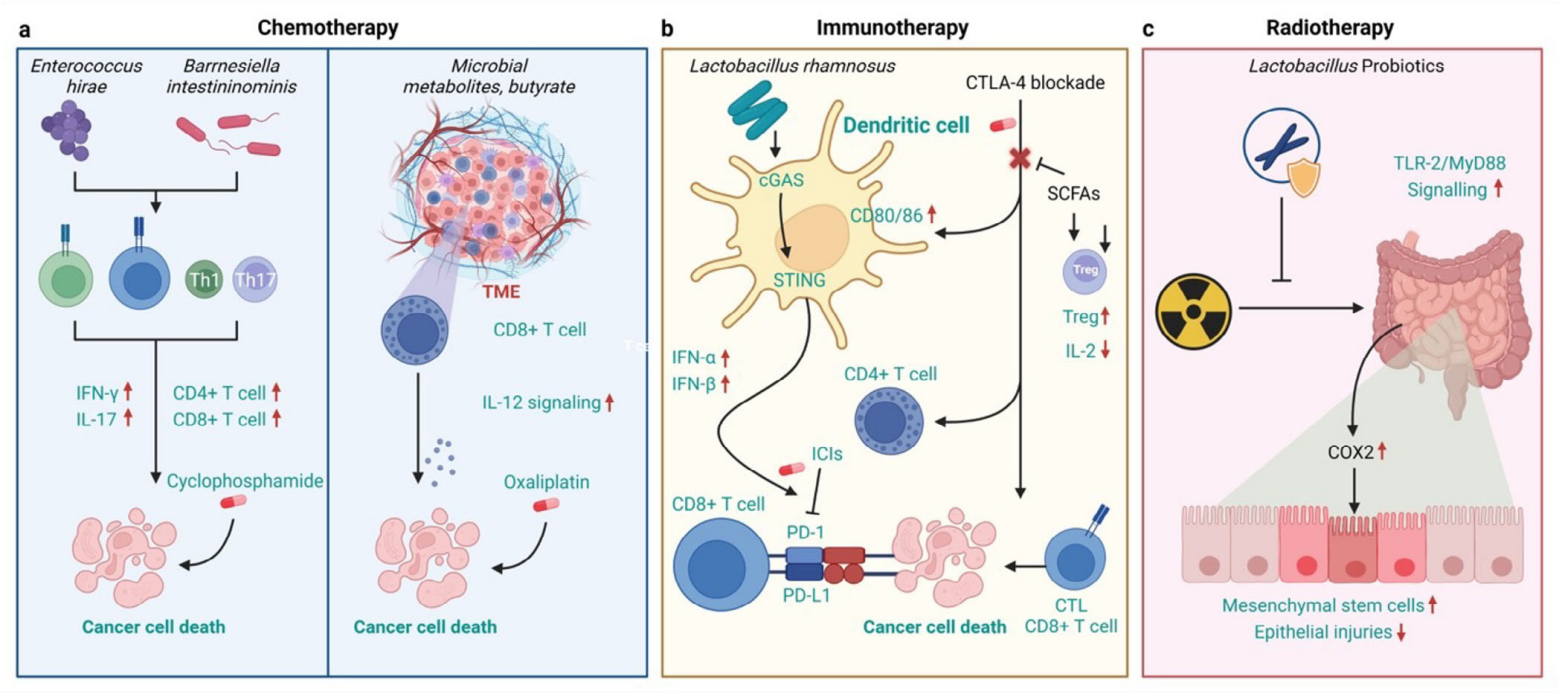
The mechanisms of microbiota impacting efficacy of cancer treatment. (A) Specifically, administration of *Enterococcus* and *Barnesiella* can restore the antitumor efficacy of cyclophosphamide‐based chemotherapy through stimulating tumor‐specific T cells and producing IFN‐γ, and butyrate, a product of dietary fiber fermented by gut microbes, can increase the anticancer effects of oxaliplatin‐based chemotherapy by regulating the function of CD8 + T cells in the TME through IL‐12 signaling. (B) *Lactobacillus rhamnosus* was illustrated to stimulate the antitumor activity of PD‐1 immunotherapy through cGAS–STING signal pathway, activating IFN‐α, β signaling, and activating cytotoxic CD8 + T cells; SCFAs limit the antitumor effects of CTLA‐4 blockade via alleviating Treg cells, and higher concentration of butyrate could decrease the anticancer activity of ipilimumab by inhibiting the accumulation of related CD4 + T cells. (C) Probiotics can protect gut mucosa from radiation injury through a TLR‐2/COX‐2‐dependent manner, stimulating mesenchymal stem cells to the crypt. FMT, fecal microbiota transplantation; SCFAs, short‐chain fatty acids; IL, interleukin; IFN‐ γ, interferon γ; CTLA‐4, cytotoxic T lymphocyte‐associated antigen 4; Treg, cell regulatory T‐cell; TLR, Toll‐like receptor; COX‐2, cyclo‐oxygenase‐2. Figure reproduced with permission and with slight modification from Ref. 357.

It is noteworthy to mention that optimum microbiome composition greatly improved RT efficacy in LC. Fecal microbiota transplantation (FMT) attenuates radiation pneumonia, valeric acid (microbiota‐derived SCFA) prolongs the survival rate, promotes hematopoietic function, and improves gut's epithelial integrity of irradiated mice.^285^, ^286^ Lung tissue protective functions of FMT fundamentally based on its gut microbiota restructuring ability that directly influence the cellular inflammatory response and oxidative stress. Intervention of FMT after local chest irradiations on mice model attenuated the lung coefficients, increased respiratory quotient value, reduced the volume of oxygen inhalations while preserving the volume of carbon dioxide exhalation constant. More importantly FMT suppressed the lung inflammation by downregulating the expression of IL‐18 and potentiate the oxidative stress scavenging system.^287^ Upon investigation of four most targetable metabolites obtained from gut microbiota metabolome, that is, trimethylamine‐N‐oxide, histidine hydrochloride hydrate, micronomicin, and prostaglandin F2α (PGF2α), PGF2α has been found to be profoundly effective to protect healthy lung cells.^288^ Dynamic GLA attributes butyrate, different gut‐derived inflammatory macrophage precursor molecules and immune cells into respiratory circulation and improve hematopoietic function.^289^, ^290^ A study done by Xiao et al.^291^ and team revealed the bone marrow and GI tract protective effect of indole 3‐propionic acid in either gender of irradiated mice along with lower incidence of systemic inflammatory reaction, which also supports the strong connection between phenotypic composition of host's microbiota and RT. Additionally, intestinal microbiome‐derived PGF2α exhibits good protective behavior to normal pulmonary cells and patients having increased level of PGF2α expression parallel to their transcription genes led to improved survival rates. Also, PGF2α‐treated irradiated mice were observed with better pulmonary ventilation and alveolar integrin where FMT‐administered mice showed less IR‐induced chest toxicity, improved lung's inflammatory status, and lower oxidative stress.^285^ Gut flora‐derived PGF2α further defends the radiation‐induced apoptotic cells deaths of normal LC by upregulating the FP/MAPK/NF‐κB signaling axis.^285^ The mechanisms of microbiota impacting efficacy of cancer treatment are shown in Figure 6.

### 4.3 Chemotherapy and microbiome

Among all the available therapeutic approaches, chemotherapy still holds the ultimate treatment option for all cancers. Started with modest clinical outcomes in LC treatments, history of chemotherapy discovered thousands of chemotherapeutic agents and keep itself being progressive with significantly improved overall survival rate of cancer patients. More recently, chemotherapy‐based combinational strategies with other anticancer therapy, such as TKIs or ICIs, have been reported with better clinical prognosis.^292^ Emesis, alopecia, infertility, and high risk of cardiac toxicity are the obvious and well‐documented adverse events associated with chemotherapeutic agents.^293^

Beside these well reported and commonly observed adverse effect, long‐term administration of chemotherapeutic agents is also reported to have host's microbiota influencing potentials which can alter the epithelial barrier integrity and mucosal homeostasis.^294^ Chemotherapy not only witnessed microbial dysbiosis, but microbial status is equally responsible for patient's responsiveness toward drugs and adverse events prevalence. Co‐administration of probiotics with chemotherapeutic agents to maintain microbiome homeostasis has been assumed to mitigate their unwanted ADR and to enhance the therapeutic efficacy.^295^ Low incidence of chemotherapy‐induced intestinal dyspepsia by *Bacillus subtilis*, inhibition of chemotherapeutics‐induced diarrhea, and systemic inflammation by *Clostridium butyricum* are some evidences supporting this hypothesis.^296^, ^297^ A study done by administering combined cisplatin and *Akkermansia muciniphila* in LC mice group resulted in small tumor volume, decreased Treg ratio, reduced Fas ligand protein expression level, and upregulated antitumor IFN‐γ, IL‐6, and TNFα compared with cisplatin only administered mice group.^298^ Co‐administration of bioengineered *Bacillus subtilis* strain designed to produce pyridoxine that stimulate the proapoptotic effect of cisplatin has been considered as one of the promising clinical approaches.^299^ Similarly, co‐administration of *Enterococcus hirae* and *Barnesiella intestinihominis* was found to be highly beneficial in advanced LC cases as it greatly empowers the immunomodulatory potency of cyclophosphamide, originally an alkylating agent. Stimulation of INF‐γ secretions from Th1 cells established a protective hallmark to the cohort under cyclophosphamide applied chemotherapy intervention.^232^ The impact of inhabited microbial species on an individual's sensitivity toward chemotherapeutic agents attracts the new researcher's concern to develop more reliable and personalized therapeutic regimens.^300^ For instance, patients with abundant *Streptococcus mutans* respond excellently toward chemotherapeutic drugs, whereas *Leuconostoc lactis* and *Eubacterium siraeum* abundant patients did not have satisfying sensitivity.^301^ Similarly, gastrointestinal reaction sensitivities induced by platinum‐based chemotherapeutic agents are highly dependable on an individual's gut microbiota phylogeny. An enteric systems with a higher relative abundance of *Bacteroides, Proteobacteria*, or *Chlamydiae* were found to be more prone toward platinum‐based chemotherapeutics‐induced gastric reaction, whereas *Firmicutes*, *Actinobacteria, Euryachaeota*, or *Fusobacteria* abundant cases were observed with lower chances of gastric reactions.^302^

### 4.4 Immunotherapy and microbiome

Extensive investigations on principles of immune responses in cancer broaden the understanding of the physiological involvement of the immune system in tumorigenesis and discover new horizons to develop immunotherapy for cancer treatment.^6^, ^24^ Tumor‐specific peptide or DNA vaccination, adoptive cell therapy, genetically engineered CAR T therapy, T cell receptor modifications, application of oncolytic virus to stimulate host's immunity, tumor antigen targeting antibodies, inhibition of immune checkpoint, and so on are some of the effective approaches of immune‐based therapeutic strategies.^7^ A wide range of cancer cells has different inhibitory immunoreceptors, namely, PD‐1, CTLA‐4, LAG3, TIM3, TIGIT, BTLA, and so on, which are named as “immune checkpoint” and understanding of their dynamic role in cancer physiology subsequently brought to the discovery of new ICIs that radically revolutionize therapeutic battel of LC, especially NSCLC.^303^, ^304^ Since immunotherapy was found to produce durable therapeutic responses as they reflect the body's endogenous immunity, newer research approaches are highly focused on developing advanced immunotherapy‐based treatment alternatives. However, with the involvement of complex cellular reactions, differences in an individual's immune status and lack of absolute biomarkers, it is difficult to measure therapeutic outcomes of immunotherapy.^305^

Emerging evidence signifies the close association between the microbiota composition of the gut and ICI therapy. Common genomic characteristics of gut microbiome readily influences the ICI efficacy and number of studies indicate that complete gut microbial composition is an essential component in improving NSCLC prognosis and treatments. Blood samples from peripheral sites of patients having optimum gut microbiota have been found to have enhanced memory T cell and NK cell signature.^306^, ^307^ Similarly LC patients with higher β‐diversity responded well to PD‐1 antagonist and *Parabacteroides* and *Methanobrevibacter* have been predicted for better LC control.^308^ Use of antibiotics attributable to cause host microbiome dysbiosis and dramatic reduction in beneficial flora subpopulations in patients under immunotherapy has been shown to impair the therapeutic efficacy.^306^, ^309^ Contrast to this, co‐administration of probiotics favored the improved clinical outcomes in advance and recurrent NSCLC receiving anti‐PD1 monotherapy. Similarly, a combination of ICIs and probiotics in NSCLC patients has been observed with the dramatic extension of the overall survival period and a significantly longer progression‐free survival rate.^241^, ^310^ Several studies suggest that diverse species of gram‐positive and gram‐negative bacterial phyla like *Bifidobacteria, Lactobacillus sp, Akkermansia muciniphila, Firmicutes*, and *Actinomyces* were closely associated with immuno‐therapeutic effectiveness.^311^ The complex mechanistic interplay between tumor suppressor *Bifidobacterium* and ICI efficacy rejects the hypothesis of the direct involvement of these bacteria in tumor suppression. Instead, it should generate specific immune cell mediated immune modulations to improve the functions of tumor‐specific CD8+ T cells. For instance, a study including the administration of *Bifidobacterium* and anti‐PD‐L1 to the CD8+ T cell knockout mice model reported that there were no synergistic effects of combination therapy in reducing the tumor volume.^312^ It has been suggested that *Bifidobacterium breve* may be considered as a potential biomarker to predict the clinical outcomes of anti‐PD‐1 treatment combined with chemotherapy as its gut richness significantly increased the progression‐free survival period of NSCLC patients. Moreover, *Bifidobacterium breve*‐rich group showed dramatically better clinical responses than the less abundant group.^313^ Interestingly, *Lactobacillus rhamnosus* was not directly involved itself, but it promotes other beneficial intestinal bacteria, which ultimately enhances the efficacy of anti‐PD‐1 therapy.^314^ It is also capable to enhance the efficient delivery of CRISPR/Cas9 via probiotic‐based self‐driven nano‐carrier system to the hypoxic region of cancer cell and induce ROS generation leading to immunogenic cell death.^315^

### 4.5 Probiotics potentially improve the immune response via gut–lung axis

Probiotics are the viable microorganisms, which when administered in adequate dose confer the health benefits of the host and have long been well known for microbiome and immune modulatory properties.^316^ Regulation of pulmonary homeostasis by probiotic supplements underlies the hypothesis of lung immunomodulatory regulation by inducing beneficial flora via GLA cross‐talk. Though GLA modulation by probiotics is gut–lung microbial context dependent and applied strain specific, colonization and maintenance of integrity of respiratory intestinal mucosa, SCFA and antipathogenic peptide production, stimulation of innate and adoptive immunity are the common hallmarks.^317^, ^318^ As previously mentioned, probiotics have been found to be highly beneficial when used in combination with various conventional LC therapies. However, since the direct effects of the probiotics on LC suppression remain unclear, more comprehensive and strain‐specific investigations are needed to elucidate the exact mechanism. Their potential to effectively modulate both local and systemic mucosal immunity in host is considered to enhance the therapeutic outcomes of the alkylating agents and immune‐modulating therapies in LC.^319^ *Lactobacillus rhamnosus, Clostridium butyricum, Bifidibacterium longum, Saccharomyces cerevisae, Akkermansia muciniphila*, and so on are some recombinant probiotics that have been well elucidated for their immune homeostasis regulatory properties in lung.^320^, ^321^, ^322^, ^323^, ^324^, ^325^, ^326^ Following the oral administration of commercially available *Bacillus subtilis* to piglet, its immune boosting potential has not been only limited to the increments of IgA secreting cells and CD3^+^ T cells count at intestinal mucosa but also at respiratory tract. It upregulated the expression of IL‐1β, IL‐5, IL‐6, TNFα, B cell activating factor and IgA promoter protein at transcriptional level throughout the lungs and respiratory tract.^327^ Similarly, administration of aerosolized *Lactobacillus rhamnosus* has been reported for its antitumor potential via maturation of alveolar macrophages. When instilled to the B12 tumor cell‐induced mice model, it amplified the maturation rate of alveolar macrophages specifically CD103+ DC and CD11b+ DC that dived to migration toward lymph nodes causing tumor‐derived antigen presentation.^328^ This macrophage maturations is not confined to tumorigenic model and antigen presenting cell specificity as same trend has been also observed in tumor‐free C57B/L mice model.^207^

Commensal bacterial supplements regulate the immune system in either way among T cell‐mediated immune response, regulation of pattern recognition receptor induced anti‐inflammatory immune cross‐reaction and secondary metabolites triggered systemic immune regulation.^329^, ^330^ They can also interact with pattern recognition receptor of innate immune cells and exposed DC to promote potent antitumor Th1 and CD8+ T cell responses. *Lactobacillus*‐stimulated human myeloid dendritic cells demonstrated the upregulation of activation and maturation markers like MHC‐II, CD‐83, CD‐80, CD‐40, and CD‐86 on their surface. Further, bacteria‐triggered DC secreted IL‐12, IL‐18, and IFN‐γ, while skewed CD4+ and CD8+ T cells to Th1 and Tc1 polarization. Th1 secretes cytokines like IFN‐γ and attributes strong anticancer effect by inducing cytotoxicity to cancer cells, inhibit angiogenesis and antigen presentation.^331^ *E. coli*‐derived LPS has been reported to be directly involved in priming of CD8+ cytotoxic T cells, promotes the IL‐12, and represses the IL‐10 expression.^332^ Hua et al. stimulated DCs with combined mixture of probiotics (*Bacillus mesentericus, Clostridium butyricum*, and *Enterococcus faecalis*) and detected upregulated antigen presentation and activation marker and increased IL‐12 production and IFN‐γ accumulation.^333^ In summary, when taking these evidence, it is obvious that certain beneficial probiotics are capable of eliciting the anticancer effect via microbiota‐mediated immune modulation at least on in vitro set‐up. Further clinical and in vivo investigation can bring their exact mechanism in human immune system and explore the potential therapeutic applications. The influence of microbiome in the treatment of LC has been summarized in Table 2.

**TABLE 2:** Influence of microbiome in the treatment of lung cancer.

Treatment intervention	Microorganism source	Experiment model	Result	References
Cisplatin	*Lactobacillus acidophilus*	Antibiotics cocktail induced dysbiosis mice.	Decreased tumor volume Longer survival times Upregulation of CD8+ T cells gene expression	^295^
Nivolumab, pembrolizumab and atezolizumab	*Clostridium butyricum*	Retrospective clinical evaluation	Increased overall survival periods Decreased GI motility related adverse events	^334^
Platinum‐based doublet chemotherapy	*Bifidobacterium lactis, Lactobacillus acidophilus*, and *Lactobacillus rhamnosus*	Randomized double blind placebo control clinical trial	Improved overall quality of life Decreased chemo‐induced pain Decreased prevalence of GI‐related complications	^335^
Recombinant sFlt‐1 gene therapy	*Bifidobacterium infantis*	Lewis's LC mice model	Enhances the targeted delivery of sFlt‐1 gene to specific tumor site Reduced tumor size Prolonged overall survival period	^336^
Virus infected erythrocytes	*Plasmodium falcifarum*	Lewis's LC mice model	Suppressed tumor growth Reduced rate of distant metastasis	^337^
Bacteria‐associated antigen gene sHSP	*Lactobacillus plantarum*	Lewis's LC mice model	Reduced tumor growth rate and tumor volume Enhanced NC8‐sHSP colonization and invasion into intestinal epithelium Promote plasmid delivery and induces endogenous plasmid expression Promote cellular immunity	^338^
Bacteria‐derived lipopolysaccharides	*Bacteroides vulgatus*	A549 LC cell line and male C57BL/6 mice	Abolished EC‐LPS induced A549 cells elongation Suppressed EC‐LPS upregulated IL‐B, IL‐6, and TNFα gene expression Reduced EC‐LPS induced lung index in vivo Prevent in vivo lung injury and inflammations	^339^

## 5 CHALLENGES ASSOCIATED WITH RESEARCH EXPLORING THE INFLUENCE OF GUT AND LUNG DYSBIOSIS ON LC PROGRESSION

Despite of evolution of selective kinase inhibitor and anti‐PD1 immune check point inhibitor along with increased 5‐year survival rate, LC still hit the major cause of all cancer death.^340^ Microbiome–host immunogenic interaction, the influence of microbial metabolomics on host physiological processes and environmental factors determines the host lung homeostasis. As microbiota are actively involved in the host immune modulation and their remarkable potential to improve conventional LC treatment, they can be considered the perfect target for fostering new therapeutic approaches.^101^, ^197^, ^290^, ^341^ Instead of much evidence explaining the direct involvement of microbiome in the inception and progression of numerous lung ailments, their host‐dependent variable composition and dynamic signaling phenotypes make it difficult to reveal the exact mechanisms.^342^, ^343^ Interpersonal arbitrariness in terms of alpha and beta diversity and microbiome‐specific immune responses make their analysis more sensitive, thus making difficulty in establishing causality pose significant challenges.^344^ Since, a biological load of the human microbiome can be extensively affected by geographical location and their biomass is highly site specific in the host body system, obtaining an accurate clinical result is quite invasive and challenging. Additionally, standardizing methods for sample collection, storage, and analysis is crucial but can be difficult to achieve across different research studies.^345^, ^346^, ^347^ Similarly, numerous confounding variables, such as diet, lifestyle, medication use, and comorbidities, can influence both the microbiome and cancer progression.^348^, ^349^ Controlling for these factors or conducting adequately powered studies to account for them is essential but can be challenging. Ethical considerations also set strict regulations in clinical settings. Conducting interventional studies to manipulate the microbiome raises ethical concerns, particularly regarding potential risks to participants and the unknown long‐term consequences of such interventions.^350^ Though animal models offer a controlled environment to study the GLA, there may be numerous limitations to fully recapitulate the complexity of human physiology and disease progression. Translating findings from animal studies to human clinical applications requires careful consideration and validation.^351^, ^352^ Validated cohort‐based controlled longitudinal studies should be designed to overcome the above‐mentioned limitations. Long‐term follow‐up studies are necessary to understand how changes in the gut and lung microbiomes over time relate to LC progression. However, such studies can be resource‐intensive and challenging, particularly in human populations. Addressing these limitations will require interdisciplinary collaboration, innovative research methodologies, and advances in technology for microbiome analysis and manipulation. Despite these challenges, investigating the role of gut and lung dysbiosis in LC progression holds promise for identifying novel diagnostic and therapeutic strategies.

## 6 CONCLUSIONS

The involvement of the gut and lung microbiomes in LC progression is supported by extensive clinical and experimental evidence. Dysbiosis in these microbiomes, the presence of specific microbial species within the LC TME, and the influence of microbial metabolites on inflammation and immune responses underscore the significance of the GLA in LC. Microbiome alterations during LC may exacerbate inflammation and promote carcinogenesis. Probiotic co‐therapy could help restore microbiome balance and improve prognosis. To validate the gut–lung microbiome as a potential LC biomarker, comprehensive, large‐scale studies are needed to explore the underlying molecular mechanisms.

## AUTHOR CONTRIBUTIONS

Keshav Raj Paude and Rajan Thapa conceptualized the review. Rajan Thapa, Keshav Raj Paude, Anjana Thapa Magar, Jesus Shrestha, Tayyaba Sadaf, and Sobia Idrees wrote the manuscript. Anjana Thapa Magar and Jesus Shrestha drew the figures using BioRender.com. Vrashabh V. Sugandhi, Ram Nikhate, Nisha Panth, Kamal Dua, Bassma H. Elwakil, Satish Rojekar, Gaurav Gupta, Sachin Kumar Singh, and Philip M Hansbro reviewed, edited, and proof read the paper. Keshav Raj Paude supervised the entire process. All authors participated in review editing and proof reading. All authors read and approved the final manuscript.

## CONFLICT OF INTEREST STATEMENT

All authors declare no conflict of interest.

## ETHICS STATEMENT

Not applicable.

## Supporting information



Supporting Information

## Data Availability

Not applicable.
