# Supplementary material for: Influence of gut and lung dysbiosis on lung cancer progression and their modulation as promising therapeutic targets: a comprehensive review
Source: MedComm (2020). 2024 Nov 24;5(12):e70018. doi: 10.1002/mco2.70018 (PMC11586092; doi:10.1002/mco2.70018)
Supplement: Supplementary file 1 — Supporting Information [file MCO2-5-e70018-s001.docx]

**Influence of gut and lung dysbiosis on lung cancer progression and their modulation as promising therapeutic targets: A comprehensive review**

Rajan Thapa^1^, Anjana Thapa Magar^2^, Jesus Shrestha^3^, Nisha Panth^4^, Sobia Idrees^4^, Tayyaba Sadaf^4^, Saroj Bashyal^5^, Bassma H. Elwakil^6^, Vrashabh V. Sugandhi^7^, Satish Rojekar^8^, Ram Nikhate^9^, Gaurav Gupta^10,11^, Sachin Kumar Singh^12,13^, Kamal Dua^13,14^, Philip M Hansbro^4,*^, Keshav Raj Paudel^4,*^

*Correspondence

Dr. Keshav Raj Paudel and Prof. Philip M. Hansbro

Centre for Inflammation, Centenary Institute and University of Technology Sydney, Faculty of Science, School of Life Sciences, Sydney, New South Wales, Australia

Email: [Keshavraj.paudel@uts.edu.au](mailto:Keshavraj.paudel@uts.edu.au), Philip.hansbro@uts.edu.au

Supplementary Figure S1 and S2


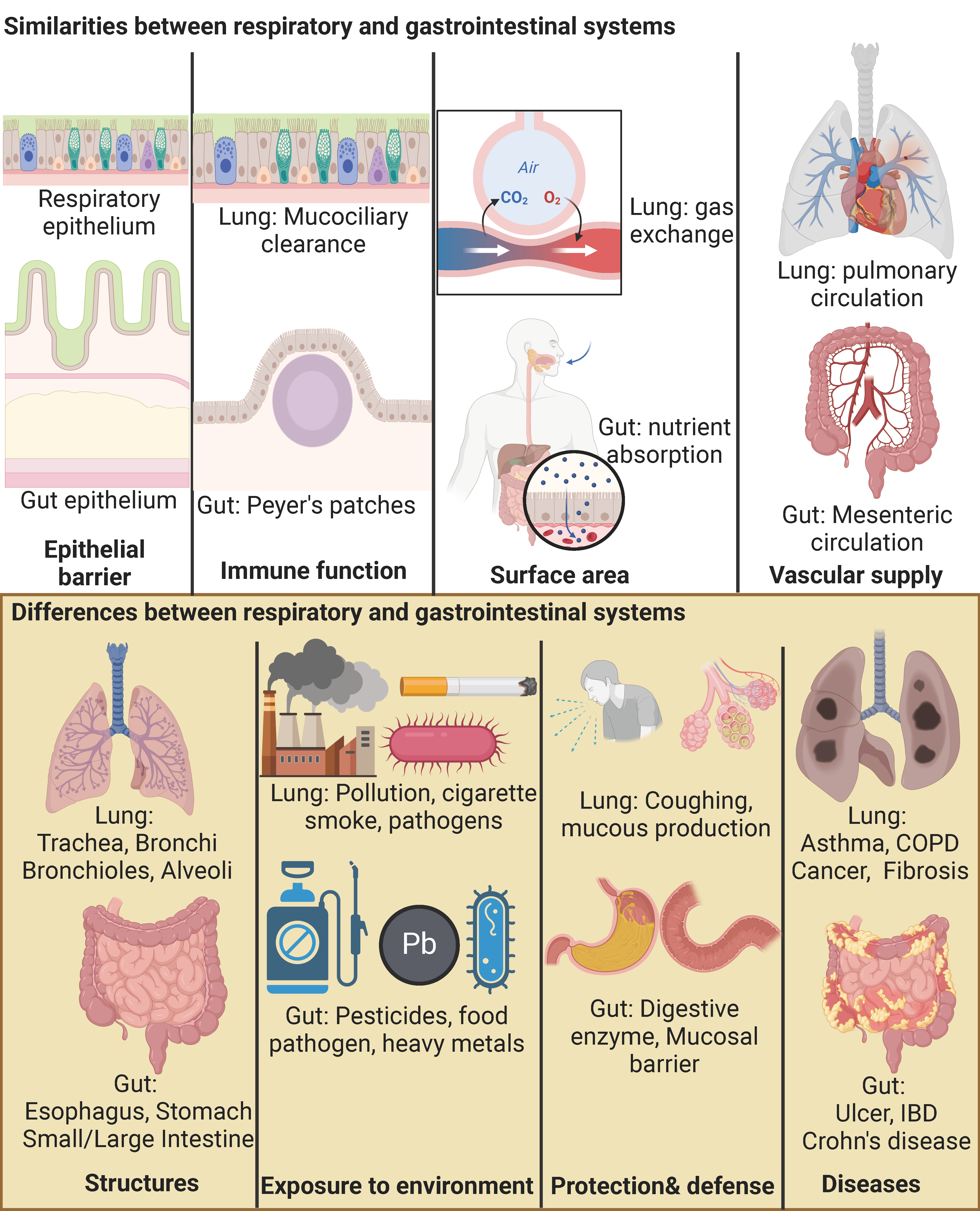


Figure S1 Structural and functional comparison of gut and lung system

Anatomically, both systems are lined with epithelial cells that act as a barrier to protect underlying tissues and regulate the exchange of substances. Presence of large number of immune cells and structure like payer’s patches in gut and mucociliary mechanism of respiratory airways bridge both organ in body’s immune defence system. Extensive alveolar surface area serves as major gaseous exchange platform and blood purification while large intestinal surface ensures the sufficient nutrient absorption into the systemic circulation. Supply with extensive vascular system, i.e. pulmonary circulation in lung and mesenteric circulation in gut, signifies the rich blood supply to both organs and their importance. In other way, lungs and gut both have differential accessary organ connection and perform specific physiological functions. Trachea, bronchi and bronchiole are closely connected to lungs and closely regulated for efficient oxygen supply throughout the body while gut works mutually with liver and pancreas regulating food metabolism and nutrient supply. Direct exposure to external environment via continuous inhalation and exhalations makes lungs more prone to airborne pathogen and particulate. Gut can interact with ingested substances and microorganism. Respiratory system constitutes coughing system and mucus production to protect lungs by external and harmful attack whereas various digestive enzyme, mucosal barrier and GALT are the core defensive system of gut. In pathological context, asthma, COPD, pneumonia are the most common disease of lungs and IBS, Crohn’s disease and peptic ulcer are the major gut associated health issues. GALT- Gut Associated Lymphoid tissue, COPD – Chronic Obstructive Pulmonary Disease, IBS – Inflammatory Bowel Syndrome. Image was created with BioRender.com.


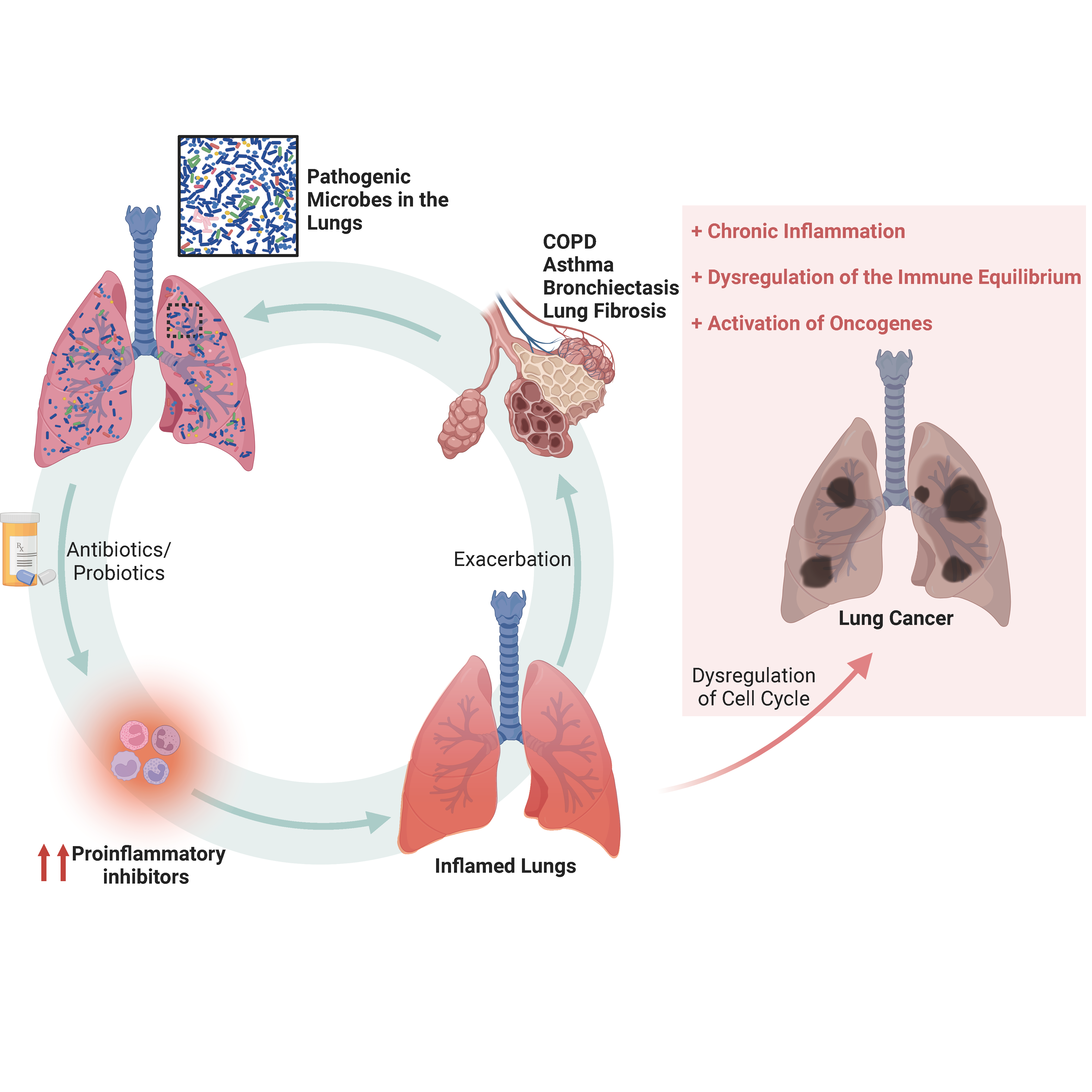


Figure S2 Role of lung microbiome in the pathogenesis of chronic lung diseases: Pathogenic microbes are predominately present in the lungs of patients with chronic respiratory diseases. This results in enhanced production of proinflammatory mediators, which eventually leads to oxidative stress induced dysregulation of cell cycle (lung cancer), or exacerbations associated with asthma/COPD. Image was created with BioRender.com.
